# Lack of effects of pioglitazone on cardiac function in patients with type 2 diabetes and evidence of left ventricular diastolic dysfunction: a tissue doppler imaging study

**DOI:** 10.1186/1475-2840-9-57

**Published:** 2010-09-23

**Authors:** Katerina K Naka, Konstantinos Pappas, Katerina Papathanassiou, Nikolaos D Papamichael, Nikolaos Kazakos, Chryssanthi Kanioglou, Demetrios Makriyiannis, Christos S Katsouras, Kostas Liveris, Agathocles Tsatsoulis, Lampros K Michalis

**Affiliations:** 1 Michaelidion Cardiac Center, University of Ioannina, University Campus, Ioannina, 45 110 Greece; 2 Department of Cardiology, University of Ioannina, University Campus, Ioannina, 45 110 Greece; 3 Department of Endocrinology, University of Ioannina, University Campus, Ioannina, 45 110 Greece; 4 Department of Endocrinology, Hatzikosta General Hospital, Makrygianni Avenue, Ioannina 45 001, Greece

## Abstract

**Background:**

Thiazolidinediones, used for the treatment of patients with type 2 diabetes mellitus (DM2), are associated with an increased incidence of heart failure. We sought to investigate the effects of pioglitazone on novel echocardiographic indices of left ventricular (LV) diastolic function in DM2 patients with LV diastolic dysfunction (LVDD).

**Methods:**

Eighty-eight asymptomatic DM2 patients on metformin and/or sulfonylureas, aged 64.5 ± 7.7 years, without known cardiovascular disease, with normal LV systolic function and evidence of LVDD were randomly assigned to pioglitazone 30 mg/day (n = 42) or an increase in dose of other oral agents (n = 39) for 6 months. All patients underwent transthoracic conventional and Tissue Doppler Imaging echocardiography at baseline and follow-up. The primary end-point was change in early diastolic velocity of the mitral annulus (E').

**Results:**

Improvement of glycaemic control was similar in the 2 groups. A significant difference (p < 0.05) between the 2 groups was found in the treatment-induced changes in fasting insulin, the insulin resistance index HOMA, HDL cholesterol, triglycerides, diastolic blood pressure (all in favor of pioglitazone) and in body weight (increase with pioglitazone). No significant changes were observed in any echocardiographic parameter in either group and did not differ between groups (p = NS for all). E' increased non-significantly and to a similar extent in both groups (p = NS).

**Conclusions:**

In asymptomatic DM2 patients with LVDD, the addition of pioglitazone to oral conventional treatment for 6 months does not induce any adverse or favorable changes in LV diastolic or systolic function despite improvements in glycaemic control, insulin sensitivity, lipid profile, and blood pressure.

## Background

Diabetes mellitus (DM) is associated with a substantially increased risk of developing heart failure (HF) [1]. Although the association between diabetes and HF has been well established, the underlying mechanisms remain vague. Co-existing morbidities, such as hypertension, microangiopathy, myocardial ischemia and renal dysfunction may explain the development of HF in type 2 DM (DM2) patients. The existence of 'diabetic cardiomyopathy', a distinct clinical process leading to HF in diabetic patients regardless of the presence of atherosclerosis and hypertension has also been suggested [2,3]. Left ventricular (LV) diastolic dysfunction (LVDD) has been recognized as an early manifestation of myocardial dysfunction in DM2 patients; LVDD assessed by echocardiography has been demonstrated in up to 60-75% of asymptomatic DM2 patients [4,5].

Pioglitazone is a thiazolidinedione used for the treatment of DM2 and acts as an insulin sensitizer [6]. Pioglitazone has also been shown to improve lipid profile, blood pressure, inflammatory biomarkers and endothelial function [7], and data suggests that it may reduce cardiovascular events in DM2 patients [8,9]. However, pioglitazone administration has been associated with an increase in the incidence of serious non-fatal HF [9,10], mainly attributed to weight gain and peripheral edema [10].

Limited evidence suggests a beneficial effect of pioglitazone on LV diastolic function in hypertensive or DM2 patients with normal cardiac function using conventional echocardiography or MRI [11,12], while pioglitazone has also been shown to prevent or improve LVDD in animal studies [13,14]. On the other hand, clinical studies have demonstrated a neutral effect of pioglitazone on cardiac structure and systolic function in DM2 patients with normal [15] or impaired systolic function [16] using conventional echocardiography. The effects of pioglitazone on echocardiographic indices in patients with DM2 and LVDD have not been largely studied, while Tissue Doppler Imaging (TDI) echocardiography that allows more direct quantification of LV diastolic function [17] has been very little used in this context [18]. In the current study, we investigated the effect of pioglitazone administration on conventional and TDI echocardiographic indices of LV function in asymptomatic DM2 patients with evidence of LVDD.

## Methods

### Study Population

Eighty-eight DM2 patients on metformin and/or sulfonylurea were enrolled in this study. Inclusion criteria were: 1) DM2 treated only with metformin and/or sulfonylurea at the time of enrolment, 2) glycaeted haemoglobin (HbA_1C_) > 6.5%, 3) evidence of LVDD on echocardiography with preserved LV ejection fraction (LVEF > 50%) and absence of any wall motion abnormality and 4) normal liver enzymes and renal function. Exclusion criteria were: 1) treatment with pioglitazone or rosiglitazone or insulin within the previous 6 months, 2) new onset of any medications within the previous 3 months, 3) any history, symptoms, signs of HF, coronary artery, cerebrovascular, or peripheral vascular disease, 4) uncontrolled hypertension, 5) atrial fibrillation, 6) more than mild valvular heart disease, 6) liver or renal disease, anaemia, thyroid dysfunction or any other major health problem and 7) diabetes-related complications (proliferative retinopathy and autonomic neuropathy).

### Study design

The study was designed as a prospective, randomized, open- label, blind evaluation with all analyses performed by personnel unaware of treatment allocation. The study evaluated echocardiographic indices of LV function in asymptomatic DM2 patients with evidence of LVDD, on metformin and/or sulfonylurea treatment, when either pioglitazone was added to their treatment or ongoing oral treatment was intensified for 6 months. The primary end-point was change in early diastolic velocity at the mitral annulus (E') from baseline to follow-up. Additional analyses included changes in glycaemic control, insulin resistance indices, lipid and blood pressure levels, LV systolic function indices, Tei index, E/A ratio, diastolic and systolic mitral annular velocities, and E/E' ratio. Patients were recruited at the Endocrinology out-patient clinics of the University and Hatzikosta General Hospitals of Ioannina from October 2004 to April 2006 after review of their medical history, a complete physical examination, an electrocardiogram and a transthoracic echocardiogram. Patients eligible for the study who consented to participate were randomly assigned in a 1:1 ratio using a computer-generated block of 4, into 2 groups. Pioglitazone (30 mg od) was added in group A (n = 44), while in group B (n = 44) an increase in the dose of the conventional medication or medications already received by the patient (metformin and/or sulfonylurea) was prescribed according to the physician's discretion in the randomization visit only. Care was taken so that the type of antidiabetic medications used in this group remained unchanged during the study (i.e. only dosage of medications could be increased and no other medication was added). Group B may be considered as a substitute for a control group to compare with pioglitazone since it was felt unethical to leave DM2 patients with established myocardial involvement without any glucose-lowering treatment.

**Figure 1:**
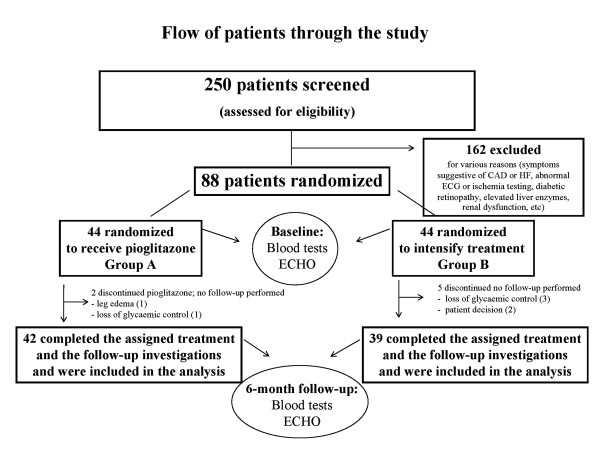
**Flow-chart of the study**. CAD, coronary artery disease; HF, heart failure; ECG, electrocardiogram; ECHO, echocardiogram.

Clinical assessment, blood sampling, and echocardiographic evaluation were performed in all patients at baseline and 6 months. All other treatment (antihypertensive, hypolipidaemic and antiplatelet) remained unaltered during the 6-month study period. Instructions were given to patients in order to maintain stable dietary habits and physical activity during the study. Liver enzymes were measured in all patients at 1, 3 and 6 months. The design of the study is presented in a flow-chart (Figure 1).

The study was approved by the Ethics Committee of the Michaelidion Cardiac Centre, University of Ioannina, Greece and written informed consent was obtained from all patients. The study was conducted in accordance with the Declaration of Helsinki.

### Laboratory investigations

Blood samples were drawn after an overnight fast for measurement of glucose, insulin, HbA_1C_, lipid concentrations, liver and renal function and full blood count using standard methods. LDL cholesterol was calculated using the Friedewald formula: LDL cholesterol = Total cholesterol - HDL cholesterol - triglycerides/5. The fasting serum glucose was divided by the fasting serum insulin to calculate the fasting glucose-to-insulin ratio, an index of insulin sensitivity. The homeostasis model assessment (HOMA), calculated as (fasting serum glucose [mmol/l] × fasting serum insulin [μU/ml])/22.5, was used as an index of insulin resistance.

### Echocardiography

Echocardiographic studies were performed using an Echo-Doppler ultrasound unit (Ultrasound ATL, HDI 5000, Bophell, WA, USA) and a 2-3.5 MHz transducer. Standard 2-dimensional (2D), 2D-guided M-mode, color flow Doppler and TDI echocardiography was performed at baseline and 6-month follow-up in all patients by one single experienced operator blinded to patients' treatment. Studies were recorded on super-VHS videotape (VCR Panasonic AG-MD 835, Osaka, Japan) for off-line analysis. Echocardiographic parameters were measured as the consensus of two experienced investigators masked to patients' treatment and study order. All recordings and measurements were obtained according to current standards of echocardiography practice [17,19].

#### Standard 2 D, 2 D guided M-mode and Doppler measurements

Standard 2 D and M-Mode measurements were obtained in the parasternal long-axis, short-axis and 4-chamber apical views and included left atrial diameter, interventricular septal and posterior wall thickness at end-diastole (IVSd and PWd respectively), LV end-diastolic and end-systolic diameter (LVDd and LVDs) and fractional shortening. LVEF was estimated using the modified Simpson's method and LV mass using the formula: LV mass = 0.8*(1.04*[(IVSd+PWd+LVDd)^3^-LVDd^3^])+0.6 [19]. LV mass was normalized for body surface area and expressed as LV mass index. LV hypertrophy (LVH) was defined as LV mass index >115 and 95 g/m^2 ^for men and women respectively [19]. Pulsed-wave Doppler was used to record blood flow velocities at the mitral inflow area. The sample volume was placed at the mitral leaflets tips in the apical 4-chamber view. Using the average of 5 beats for the analysis at a sweep rate of 100 mm/sec, the peak early diastolic and atrial filling velocity (E and A respectively) were recorded and the E to A ratio (E/A) was calculated. The E wave deceleration time was also measured. Global LV function was assessed by calculating the Tei index as previously described [20].

#### Tissue Doppler Imaging (TDI)

Mitral annular tissue Doppler velocities were recorded by pulsed-wave TDI of both septal and lateral walls in the apical 4-chamber view [17]. Signals were obtained from 3 end-expiratory cycles, and averages were made for the systolic and diastolic velocities at a sweep rate of 50 mm/sec. Tissue Doppler systolic (S') and diastolic (early, E'; late, A') velocities were recorded at the septal and lateral edge of the mitral annulus. From these, average early diastolic mitral annular velocity was calculated and the E/E' ratio was derived. The corresponding tissue Doppler velocities of the tricuspid annulus (tricuspid S', E' and A') were also obtained. To assess reproducibility, 15 patients (10 females) were studied on 2 occasions 1 week apart and the primary end-point E' re-measured by blinded investigators. A coefficient of variability (SD/mean of the differences between measurements) of 6.1% was calculated.

Simple echocardiographic measurements, described in detail above, were chosen to define LVDD. Evidence of LVDD was considered if one of the following was present: (1) E/A <0.75 or >2, (2) both septal and lateral E' <8 cm/sec, and (3) septal or lateral E'/A' ratio <1 [5,17].

### Statistical analysis

Results are presented as mean ± SD. Kolmogorov-Smirnov Z-test was used to determine the normal distribution of continuous variables: fasting insulin, glucose-to-insulin ratio and HOMA were not normally distributed. The *χ*^2 ^test and unpaired Student's *t*-test were used to compare categorical and continuous variables respectively between the 2 groups at baseline. For not normally distributed variables the Mann-Whitney test was used. Within group changes from baseline were assessed with the paired Student's *t*-test or the Wilcoxon's test. Repeated Measures Analysis of Variance (RM ANOVA) was performed to evaluate changes at follow-up between the 2 groups. Based on pilot data in DM2 patients of both genders, power calculations were performed; 40 patients were required to detect a 15% change in average E' with a power of approximately 85% for two-sided *P *= 0.05. Pearson and Spearman correlation coefficients were estimated between baseline E', as well as changes in E' at 6 months, and all relevant variables in the 2 groups. A p value of <0.05 was considered significant. The SPSS statistical software package (version 15.0 for Windows, SPSS Inc. Chicago, IL, USA) was used.

## Results

Eighty-one patients (group A, n = 42 and group B, n = 39) completed the study (assigned treatment and follow-up investigations) and were included in the analysis (Figure 1). Two patients in group A discontinued pioglitazone; one developed leg edema and one needed insulin for glycaemic control. Five patients in group B were withdrawn from the study; 3 needed insulin and 2 no longer wished to participate. Pioglitazone treatment was not associated with liver enzyme abnormalities. No other symptoms or signs of HF or other cardiovascular adverse events were observed during the 6-month follow-up.

Demographic and clinical characteristics of the 81 patients are shown in Table 1. The two groups consisted predominantly of women (64 and 72% respectively, P = NS) and did not differ in age or any other characteristic (Table 1). All female study participants were postmenopausal and did not receive hormone replacement therapy. Physical and laboratory measurements for both groups at baseline and 6 months are shown in Table 2. The two groups did not differ in any parameter at baseline.

**Table 1:** Baseline demographic and clinical characteristics in the 2 groups

Variable	Group A Pioglitazone (n = 42)	Group B Other treatment (n = 39)	P value
Age, years (range)	64.3 ± 8.1 (44-77)	64.8 ± 7.5 (48-81)	0.78

Female gender, n (%)	27 (64)	28 (72)	0.49

Hypertensives, n (%)	31 (74)	32 (82)	0.27

Hyperlipidaemics, n (%)	26 (62)	27 (69)	0.64

Ever smokers, n (%)	14 (33)	10 (26)	0.48

Family history of CAD	23 (55)	24 (62)	0.35

Duration of diabetes, years	10.2 ± 6.6	9.0 ± 6.9	0.41

Metformin	31 (74)	26 (67)	0.63

Sulfonylurea	31 (74)	27 (69)	0.81

Metformin & Sulfonylurea	20 (48)	15 (38)	0.50

Antihypertensives, n (%)	27 (64)	27 (69)	0.81

Diuretics, n (%)	6 (14)	8 (21)	0.56

ACE or AT-II inhibitors, n (%)	22 (52)	22 (56)	0.82

β-blockers, n (%)	1 (2)	3 (8)	0.35

Calcium channel blockers, n (%)	12 (29)	11 (28)	1.00

Statin, n (%)	18 (43)	17 (44)	1.00

Antiplatelets, n (%)	5 (12)	4 (10)	1.00

Mean ± SD unless otherwise stated.

CAD, coronary artery disease; ACE, angiotensin converting enzyme; AT-II, angiotensin II.

**Table 2:** Physical and laboratory measurements in the 2 groups at baseline and 6-month follow-up.

Variable	Group A, Pioglitazone (n = 42)			Group B, Other treatment (n = 39)			Comparison of changes between the 2 groups
	**Baseline**	**Follow-up**	**P value**	**Baseline**	**Follow-up**	**P value**	**P value (RM ANOVA)**

Body weight, kg	76.6 ± 12.5	78.8 ± 12.9	**0.025**	74.2 ± 10.8	74.2 ± 11.7	0.50	**0.026**

Waist, cm	104.7 ± 11.2	104.0 ± 12.0	0.12	105.6 ± 15.9	104.0 ± 10.2	0.63	0.19

Heart rate, beats/min	73 ± 11	72 ± 11	0.58	73 ± 8	71 ± 10	0.08	0.86

Systolic BP, mmHg	143 ± 15	141 ± 15	0.47	150 ± 17	146 ± 18	0.21	0.62

Diastolic BP, mmHg	78 ± 6	75 ± 7	**0.003**	78 ± 8	79 ± 9	0.64	**0.024**

Total cholesterol, mg/dl	214 ± 40	224 ± 45	**0.000**	212 ± 43	226 ± 44	**0.019**	0.45

Triglycerides, mg/dl	142 ± 60	134 ± 51	0.31	143 ± 94	160 ± 80	**0.050**	**0.030**

HDL cholesterol, mg/dl	53 ± 11	56 ± 12	**0.002**	56 ± 14	53 ± 12	**0.017**	**0.014**

LDL cholesterol, mg/dl	134 ± 33	141 ± 39	**0.018**	125 ± 44	141 ± 39	**0.017**	0.22

Fasting glucose, mg/dl	154 ± 31	146 ± 31	**0.017**	153 ± 33	149 ± 29	0.23	0.37

Fasting insulin, μU/ml	9.9 ± 5.2	7.7 ± 3.3	**0.000**	9.3 ± 4.8	10.2 ± 5.3	0.42	**0.006**

HbA_1C_, %	8.0 ± 0.9	7.5 ± 1.0	**0.000**	7.9 ± 1.0	7.5 ± 1.3	**0.002**	0.28

Glucose-to-insulin ratio	19.0 ± 10.3	23.5 ± 13.6	**0.000**	21.4 ± 15.1	22.1 ± 21.1	0.51	0.08

HOMA	3.9 ± 2.6	2.7 ± 1.2	**0.000**	3.5 ± 1.8	3.6 ± 1.8	0.38	**0.001**

Mean ± SD. * *p *< 0.05 compared to group A

BP, blood pressure; HDL, high density lipoprotein; LDL, low density lipoprotein; HbA_1C_, glycaeted hemoglobin; HOMA, homeostasis model assessment; RM ANOVA, repeated measures analysis of variance.

In both treatment groups, a similar significant reduction in HbA_1C _(by 0.57 ± 0.71% and 0.37 ± 0.91% in groups A and B respectively, p = NS between groups) and increase in total and LDL cholesterol (p < 0.05 for both, p = NS between groups) was observed at 6 months. In group A, pioglitazone also induced a significant increase in body weight, HDL cholesterol levels and glucose-to-insulin ratio and a significant reduction in fasting glucose and insulin, HOMA, and diastolic BP (p < 0.05 for all). A significant decrease in HDL cholesterol (p < 0.05) was observed in group B (Table 2). Using RM ANOVA, a significant difference (p < 0.05) was found between the 2 groups in the treatment-induced changes in: body weight, diastolic BP, triglycerides, HDL cholesterol, fasting insulin and HOMA. Changes in other parameters at follow-up did not differ between the groups.

### Echocardiographic observations

By study protocol, all patients enrolled in the study had preserved LV systolic function and echocardiographic evidence of LVDD as defined above. No patient had a restrictive pulsed Doppler pattern (E/A >2). LVH was present in 29 patients in each group (69% and 74% in groups A and B respectively, p = NS). No significant differences were observed between the 2 groups at baseline in any conventional or TDI echocardiographic measurements apart from deceleration time that was significantly lower in group A (p = 0.04) (Table 3). Baseline E' correlated inversely with age (r -3.87, p = 0.001) and presence of hypertension (r -0.359, p = 0.002) in all patients.

**Table 3:** Echocardiographic measurements in the 2 groups at baseline and 6-month follow-up.

Variable	Group A, Pioglitazone (n = 42)			Group B, Other treatment (n = 39)			Comparison of changes between the 2 groups
	**Baseline**	**Follow-up**	**P value**	**Baseline**	**Follow-up**	**P value**	**P value (RM ANOVA)**

**CONVENTIONAL ECHOCARDIOGRAPHY**							

Left atrial diameter, mm	39.5 ± 6.1	39.4 ± 5.5	0.84	37.9 ± 5.2	39.1 ± 5.3	0.15	0.22

IVSd, mm	11.4 ± 2.1	11.0 ± 2.0	0.23	11.8 ± 1.8	11.7 ± 2.2	0.79	0.58

PWd, mm	10.7 ± 1.8	10.3 ± 1.8	0.23	11.1 ± 1.6	10.7 ± 1.5	0.14	0.99

LVDd, mm	50.0 ± 5.4	49.6 ± 5.6	0.52	48.6 ± 4.4	47.3 ± 5.7	0.20	0.44

LVDs, mm	29.4 ± 5.8	30.2 ± 4.3	0.31	28.7 ± 4.1	28.8 ± 5.6	0.97	0.49

LV mass index, g/m^2^	113.8 ± 27.9	107.8 ± 33.3	0.21	118.1 ± 28.9	110.2 ± 28.2	0.17	0.78

Fractional shortening, (%)	41.4 ± 6.2	40.2 ± 6.1	0.17	41.0 ± 6.5	39.2 ± 8.1	0.14	0.69

Ejection Fraction, (%)	71.4 ± 7.1	69.7 ± 7.2	0.10	71.0 ± 7.7	68.9 ± 9.6	0.13	0.77

E, cm/s	66.1 ± 15.9	66.2 ± 15.5	0.94	62.6 ± 15.4	6.3 ± 14.0	0.26	0.42

A, cm/s	79.6 ± 19.7	81.3 ± 23.7	0.42	80.2 ± 19.6	82.8 ± 14.6	0.23	0.76

E/A ratio	0.87 ± 0.27	0.86 ± 0.23	0.72	0.80 ± 0.20	0.80 ± 0.15	0.88	0.87

Deceleration Time, ms	193 ± 43	194 ± 49	0.88	215 ± 54 *	230 ± 41	0.10	0.16

Tei index	0.35 ± 0.15	0.35 ± 0.22	0.91	0.38 ± 0.18	0.35 ± 0.15	0.37	0.52

**TISSUE DOPPLER IMAGING ECHOCARDIOGRAPHY**							

Septal S', cm/s	7.9 ± 0.9	8.1 ± 1.1	0.27	7.8 ± 1.2	7.6 ± 1.3	0.59	0.26

Septal E', cm/s	6.8 ± 1.8	7.0 ± 1.7	0.46	6.7 ± 1.4	7.3 ± 2.1	0.18	0.43

Septal A', cm/s	10.5 ± 1.6	10.8 ± 1.6	0.29	10.6 ± 2.0	10.3 ± 1.9	0.34	0.15

Septal E'/A' ratio	0.67 ± 0.22	0.66 ± 0.20	0.87	0.65 ± 0.18	0.71 ± 0.17	0.14	0.23

Lateral S', cm/s	7.7 ± 1.5	8.1 ± 1.5	0.16	8.0 ± 1.7	8.0 ± 1.6	0.99	0.34

Lateral E', cm/s	8.1 ± 2.5	8.4 ± 2.5	0.39	8.3 ± 2.0	8.6 ± 3.1	0.45	0.94

Lateral A', cm/s	11.4 ± 2.4	11.5 ± 2.5	0.84	11.1 ± 2.2	10.7 ± 2.9	0.31	0.40

Lateral E'/A' ratio	0.75 ± 0.29	0.78 ± 0.38	0.53	0.79 ± 0.27	0.84 ± 0.31	0.39	0.96

Tricuspid S', cm/s	14.5 ± 2.6	15.4 ± 3.7	0.09	14.1 ± 4.0	14.4 ± 3.3	0.60	0.49

Tricuspid E', cm/s	12.2 ± 2.9	12.0 ± 3.3	0.63	11.3 ± 3.8	11.9 ± 3.0	0.34	0.29

Tricuspid A', cm/s	18.3 ± 3.7	18.1 ± 3.9	0.82	17.8 ± 4.5	18.3 ± 4.5	0.42	0.48

Tricuspid E'/A' ratio	0.69 ± 0.20	0.68 ± 0.24	0.89	0.65 ± 0.18	0.68 ± 0.17	0.36	0.59

E' (average), cm/s	7.36 ± 1.85	7.66 ± 1.87	0.30	7.55 ± 1.44	7.96 ± 2.31	0.28	0.82

E/E'	9.14 ± 2.39	9.08 ± 2.71	0.80	8.82 ± 2.61	8.68 ± 2.63	0.73	0.87

Mean ± SD. * *p *= 0.04 compared to group A.

IVSd/PWd, interventricular/posterior wall end-diastolic thickness; LVDd/LVDs, left ventricular end-diastolic/end-systolic internal diameter; E, early mitral filling velocity; A, atrial filling velocity; S', E' and A', systolic, early and late diastolic velocity respectively of the mitral annulus; RM ANOVA, repeated measures analysis of variance.

No significant changes were seen in either group in any echocardiographic measurement at 6 months (p = NS for all, Table 3). Using RM ANOVA, it was found that changes at follow-up did not differ significantly between the 2 groups (p = NS for all). Adjustment for confounding factors was not needed since no statistical difference in any baseline characteristics between the groups (p > 0.27 for all variables) was observed. More specifically, in both groups E' velocity increased (by 6.7 ± 22.7% and 6.9 ± 30.7% in groups A and B respectively) and E/E' ratio decreased at follow-up (by 0.03 ± 16.97% and 3.18 ± 34.87% respectively), but these changes were not significant within either group and did not differ between groups (p = NS for all comparisons). Changes in E' at 6 months correlated only with changes in HbA_1c _in group A (r -0.484, p = 0.0003) and with age in group B (r -0.435, p = 0.046). Finally, no significant difference in the effect of pioglitazone vs the control group B was found when gender-interaction analysis was performed or in any high-risk subgroups, i.e. aged >60 years, diabetes duration >10 years, hypertensives, patients with LVH (p > 0.05 for all comparisons, data not shown).

## Discussion

Our study indicates that the addition of pioglitazone to standard oral treatment for 6 months in asymptomatic DM2 patients with normal systolic function and evidence of LVDD, does not induce any adverse or favorable changes in conventional or TDI indices of LV diastolic or systolic function despite significant metabolic changes. Improvement in glycaemic control and insulin sensitivity, reduction in BP, increase in body weight and changes in lipid profile were observed with pioglitazone treatment.

Pioglitazone and rosiglitazone are the 2 thiazolidinediones (TZDs) currently used for the treatment of DM2. In addition to reducing blood glucose and insulin resistance, several beneficial pleiotropic effects of TZDs have been reported [6,7,21]. Large clinical studies and meta-analyses have shown that pioglitazone reduces the risk of cardiovascular events but its use is associated with development of non-fatal HF [8,9]. Rosiglitazone has also been associated with an increased risk of HF and myocardial infarction [22]. Recent data suggests that compared with pioglitazone, rosiglitazone administration was associated with an increased risk of stroke, heart failure, and all-cause mortality in patients 65 years or older [23]. The mechanisms underlying the development of HF after TZDs administration are not fully understood. The combination of fluid retention caused by TZDs and the high prevalence of LVDD in DM2 patients may explain the increased incidence of HF following TZDs administration [10]. However, TZDs may also have an effect on cardiac structure and function [11,15,16,18]. Reversible mitral regurgitation has also been reported during pioglitazone treatment [24].

Neutral effects on cardiac structure and systolic function have been reported with pioglitazone in DM2 patients with normal systolic function despite signs of volume overload and fluid retention [15,25] and in DM2 patients with pre-existing systolic dysfunction despite an increase in HF hospitalizations [16]. Similar neutral effects have been observed with rosiglitazone [26,27].

Experimental and some recent clinical studies have investigated the effect of TZDs on LV diastolic function. Pioglitazone has been shown to improve LVDD in prediabetic rats [13] or prevent LVDD in type 2 diabetic rats [14] and several mechanisms such as altered myocardial fatty acid metabolism, improved hyperglycaemia, hyperlipidaemia, oxidative stress have been implicated. Effects of pioglitazone on angiogenesis independent of glucose lowering or PPAR-γ activation may also play a role [28]. In non-diabetic hypertensive patients, pioglitazone was shown to improve conventional echocardiographic indices of LV diastolic function (increase in E/A ratio and deceleration time) [11]. In a small study in DM2 patients, rosiglitazone caused an increase in mitral annulus velocity E' (by 12.7%) indicating improved myocardial diastolic properties, with a concomitant increase in fractional shortening [29]. An increase in septal E' velocity (by 7.7%), that correlated with a decrease in plasma type III collagen, was also found following pioglitazone treatment in another small study in DM2 patients [18]. In a recent sophisticated MRI/PET study in men with DM2 and relatively normal cardiac function, pioglitazone was shown to induce slight changes in transmitral filling rate and LV diastolic volume at unchanged filling pressures, compatible with improved LV compliance [12]; this improvement could not be explained by any changes in metabolic variables or myocardial substrate metabolism.

In the present study in DM2 patients with LVDD, pioglitazone did not appear to have a significant beneficial or harmful effect on cardiac structure or function, despite the well known effects of pioglitazone on various metabolic parameters [6,7,21]. Left atrial and LV diameters and LVEF remained unchanged reflecting probably absence of significant volume overload, similar to previous reports [16,18]. LV diastolic properties were not altered; E' increased non-significantly and to a similar extent (by about 7%) in both groups. Myocardial velocities at the tricuspid annulus remained unchanged, suggesting also a neutral effect of pioglitazone on the right ventricle, which has not been previously reported.

Several reasons may explain why the use of pioglitazone in the present study was not associated with a beneficial effect on cardiac diastolic properties in contrast to some recent reports. The population included in the current study was selected on the basis of evidence of LVDD that may be attributed to various factors in these patients; apart from DM2, hypertension and ageing may also be important contributors. DM2 patients included in the study had a high incidence of hypertension that is known to further deteriorate LV diastolic function [30]; baseline E' in all patients correlated inversely with age and presence of hypertension similar to previous reports [30]. Whatever the components of LVDD in these patients, its presence suggests some myocardial involvement that may have limited the potential beneficial effects of pioglitazone on diastolic function. Increased myocardial fibrosis has been associated with LVDD and such structural changes are considered to be less amenable to treatment [31]. Furthermore, the benefit associated with the use of pioglitazone may be too small to be detected even with the use of TDI methodology that is considered superior to conventional echocardiography for the assessment of LV function [5,17,32]. In any case, great changes in myocardial diastolic properties that would be easily detected by echocardiography and would probably lead to improved clinical outcome have not been observed with 6-month pioglitazone treatment. In the present study, the increase in E' velocity with pioglitazone was associated with the improvement in glycaemic control. It may be possible that a higher dose of pioglitazone leading to better glycaemic control may have been necessary to induce significant changes. However, the extent of metabolic control amelioration has not associated with the improvement in LV diastolic function in a recent randomized trial comparing insulin to metformin/sulfonylureas [33].

### Study limitations

Although this was a single-center study, the study population provided adequate power to detect modest changes in echocardiographic indices of LV diastolic function that were estimated based on previous studies [12,18,29] and the reproducibility of our methodology. This was a randomized, open-label study, but echocardiographic recordings and measurements were performed by operators blinded to patients' treatment. Our population consisted mainly of postmenopausal females and hence the reported results may not be applicable to the general population. However, gender-interaction analysis excluded a possible gender effect in our study. Other medications received by study participants (e.g. angiotensin converting enzyme or angiotensin II inhibitors, calcium channel blockers) may have affected LV diastolic function, potentially attenuating the effect of pioglitazone; however, no changes were allowed during the study. A combination of simple and easy-to-acquire criteria was used for the detection of LVDD; their selection was based on previous publications since recommendations on diastolic function evaluation were only recently published [34]. Although these criteria are largely included in recent recommendations on LV diastolic function evaluation [34], it cannot be excluded that LVDD may be over-diagnosed in a small number of patients. More sophisticated measures such as the average diastolic velocities from all points in the mitral annulus or the left atrial volume could be used in future research to reveal more subtle changes in LV diastolic function. Only one patient developed leg edema, without any other symptoms or signs of HF, during the study in the pioglitazone group and the patient discontinued pioglitazone on his own long before any follow-up echocardiography could be performed. Finally, coronary artery disease was not vigorously excluded with angiography or non-invasive assessment of inducible ischemia; the potential presence of coronary artery disease may have affected the effect of pioglitazone on diastolic function.

## Conclusions

In asymptomatic DM2 patients with evidence of LVDD and preserved systolic function, 6-month treatment with pioglitazone has no favorable or harmful effect on echocardiographic indices of cardiac structure, systolic or diastolic function despite favorable effects on glycaemic control, insulin resistance, lipid profile and blood pressure. Although the use of TZDs has been associated with induction of HF in some DM2 patients, pioglitazone did not appear to have a harmful effect on intrinsic diastolic properties of the myocardium in DM2 patients with pre-existing LVDD. Larger studies with more sensitive methods are needed in order to reveal potential beneficial effects of pioglitazone on myocardial diastolic properties.

## Competing interests

The authors declare that they have no competing interests.

## Authors' contributions

**KKN **has contributed to (1) conception and design, (2) acquisition of data, (3) analysis and interpretation of data, (4) drafting and revising the article critically for important intellectual content. **KoP **has contributed to (1) conception and design, (2) acquisition of data, (3) analysis and interpretation of data, (4) drafting and revising the article critically for important intellectual content. **KaP **has contributed to (1) acquisition of data, (2) analysis and interpretation of data, (3) drafting and revising the article critically for important intellectual content. **NDP **has contributed to (1) analysis and interpretation of data, (2) drafting and revising the article critically for important intellectual content. **NK **has contributed to (1) acquisition of data, (2) analysis and interpretation of data, (3) drafting the article critically for important intellectual content. **CK **has contributed to (1) acquisition of data, (2) interpretation of data, (3) drafting the article critically for important intellectual content. **DM **has contributed to (1) conception and design, (2) acquisition of data, (3) interpretation of data. **CSK **has contributed to (1) conception and design, (2) interpretation of data, (3) drafting the article critically for important intellectual content. **KL **has contributed to (1) acquisition of data, (2) interpretation of data, (3) drafting the article critically for important intellectual content. **AT **has contributed to (1) conception and design, (2) interpretation of data, (3) drafting and revising the article critically for important intellectual content. **LKM **has contributed to (1) conception and design, (2) analysis and interpretation of data, (3) drafting and revising the article critically for important intellectual content.

All authors read and approved the final manuscript.
